# Issues in optimising and standardising the accuracy and utility of the colposcopic examination in the HPV era

**DOI:** 10.3332/ecancer.2015.530

**Published:** 2015-04-29

**Authors:** Mark Schiffman, Nicolas Wentzensen

**Affiliations:** Division of Cancer Epidemiology and Genetics, National Cancer Institute, NIH, USA 9609 Medical Center Drive, Rockville, MD 20850

**Keywords:** cervix, screening, colposcopy, HPV

## Abstract

For this tribute to Mario Sideri, we reviewed some of the current issues in colposcopy, many of which we were researching with him. The review concentrates on the impact of HPV testing on cervical screening, specifically on the practice of colposcopy as the major diagnostic procedure in cervical screening programmes. Topics include the changing population of women referred to colposcopy, evolving views of the colposcopic impression, differing approaches to directed and random biopsy, issues in teaching colposcopy using static images, and the development of colposcopy aids, and simplified visual assessment techniques.

Colposcopic examination and directed biopsies remain the major diagnostic procedure in cervical cancer screening. But since cervical screening is changing because of advances in the understanding of cervical carcinogenesis, a scrutiny of the practice of colposcopy in the human papillomavirus (HPV) era is therefore warranted.

Conventional cervical cancer screening programmes typically have the following steps. 1) *screening*: periodic screening tests in the general population to find women at risk; 2) initial *management of non-normal screening results*: colposcopic examination and biopsy of women with definite screening abnormalities (e.g. low- or high-grade lesion), or intensified repeated cytology/HPV testing to triage women who screen equivocally positive (e.g. ASCUS); 3) *post-colposcopic management*: treatment of precancer/cancer found on colposcopic biopsy, or intensified re-testing of women with no cancer or precancerous lesions (at least initially) found; and 4) *post-treatment management*: follow-up of treated women [1–4]. Whereas screening, triage, post-colposcopic management, and even post-treatment management protocols are evolving based on new knowledge about HPV and cervical carcinogenesis, the colposcopic examination itself has changed little. It remains a visually-guided estimation of the severity of cervical pathology, with biopsy of lesions suspected to represent cancer or its precursors. In some ways, colposcopy lags behind the HPV-related technical advances that promise to modernise cervical screening programmes. We will review some of these issues, which were of major interest to our colleague Mario Sideri to whom this manuscript is dedicated [5–10]. The comments are general, but refer especially to the US setting; thus we do not discuss topics such as screening colposcopy.

HPV testing is being increasingly introduced in primary cervical cancer screening and management of screening abnormalities [11, 12]. HPV is the required cause of almost all cervical cancers; HPV infection is the first step in the multi-step progression to cervical cancer. Therefore, HPV-based screening allows detecting lesions earlier than cytology. Because of this change in screening and triage protocols, the population of women referred to colposcopy is changing. Colposcopy was originally designed for diagnosis of cancer and severe precancerous lesions requiring treatment. These relatively uncommon, very severe lesions are almost universally HPV-positive and are evident on colposcopy, but do not constitute the majority of examinations today. More minor abnormalities form the bulk of colposcopic examinations. Many of the referrals based on the relatively common Bethesda system, i.e. results of atypical squamous cells of undetermined significance (ASCUS) or the analogous reading of borderline dyskaryosis would never progress to cervical cancer. Triage of these equivocal cytologic findings by HPV testing, to determine need for immediate colposcopy, has proved to be cost-effective. Now, however, we are entering an era when HPV testing is being used or proposed for primary screening because of its high sensitivity for the detection of cancer and precancer [13–15]. In contrast to cytology, the non-specificity of HPV testing results from positive high-risk HPV tests that are benign infections destined to clear without need for intervention. Such benign infections are even more common than equivocal cytologic findings. Thus, referring all HPV-positive women immediately to colposcopy would flood the colposcopist with unproductive examinations; hence, the even more acute need for triage in screen-positives [4]. The earlier, subtler spectrum of lesions detected by positive HPV testing as opposed to cytologic abnormalities, further challenges the visual technique of colposcopy. The best choice of triage method for HPV-positive women is not yet determined. Current guidelines in the US recommend referral to colposcopy of women who have had two consecutive positive HPV tests without cytologic abnormalities [11]. The consequence is that the practice of colposcopy has become even more challenging in the HPV era, since some early lesions that are not even detected by cytology need to be evaluated at colposcopy.

Despite a changing referred population presenting even more diagnostic challenges, the basic equipment and reagents of colposcopy are unchanged [16]. The classic equipment remains standard (we will discuss colposcopic adjuncts below). The use of acetic acid to highlight lesions is still fundamental, with some use of Lugol’s solution. Acetowhitening has been demonstrated to be sensitive for detection of cancer and precancer, i.e. serious lesions are almost always acetowhite, but the change is non-specific [17]. Less research has been conducted on the diagnostic properties of Lugol’s staining. More specific topical markers could help to highlight cervical precancers at colposcopy, but no revolutionary new stain is on the immediate horizon, although research on candidates proceeds actively.

Equipment and reagents aside, the practice of colposcopy is under revision but without a consensus on how to improve it. In discussing colposcopic practice, it is worth noting that colposcopy serves two discrete functions: estimation of the severity of underlying cervicovaginal disease (colposcopic impression) and placement of biopsies. Ultimately, the colposcopic examination results in treatment decisions, which can be based on colposcopic impression or biopsy results in the context of screening and triage test results and previous medical history. Importantly, the approach to confirming a precancer at colposcopy differs widely in different settings, suggesting that there is a lack of strong evidence for any of the approaches.

To discuss colposcopic impression first: as usually practiced, colposcopy has not proven particularly reproducible or accurate when formally evaluated. Colposcopic impression is typically derived from a criteria-based evaluation of visible lesions. The criteria used have varied. Reid’s criteria, including consideration of factors such as lesion color, margins, and aspects of vascularity have been widely taught in the U.S. [16]. The Swede score was developed to modify some of the Reid criteria and to expand the criteria to include lesion size [18, 19]. The evidence for cancer risk discrimination provided by colposcopic impression is very heterogeneous. A normal colposcopic impression, leading to no biopsies, is often taken to represent absence of cervical precancer, but the negative predictive value of this assessment is imperfect. Grading of the non-normal (e.g. low-grade versus high-grade) colposcopic impression is inaccurate and not especially reproducible.

Recently, the International Federation for Cervical Pathology and Colposcopy (IFCPC) published a new colposcopy terminology [5]. The new terminology includes several new colposcopic signs and emphasises the evaluation of lesion location that is closely linked to excision treatment types and dimensions. Formal evaluation of the reproducibility and accuracy of this new system for determining presence or risk of cancer/precancer is ongoing.

An important role of colposcopy is to guide the placement of biopsies to diagnose the underlying disease state. Histologic diagnosis of cervical disease (which itself is imperfect) invariably includes any error in biopsy placement. Biopsy protocols remain varied among colposcopists, and the choice of protocols is frankly controversial. At the simplest level, technical issues like the forceps type and biopsy size are not standardised. But the major issue by far is how many biopsies to take and how to choose their location. There are at least three approaches that are prominently advocated: biopsy of the worst-appearing lesion if a lesion is observed, biopsy of several acetowhite lesions, and strategies involving random biopsies of normal-appearing epithelium in four quadrants [20, 21]. These strategies are presented in decreasing order of confidence based on which the colposcopist can accurately determine the true worst disease visually. An important limitation of many studies evaluating the different approaches to detection of cervical precancers is the problem of verification bias: For example, if a study evaluates taking a single biopsy only, i.e. only evaluates disease endpoints based on the single biopsy, disease missed by this strategy cannot be detected, unless there is extended follow-up. As a result, the performance of a single biopsy to detect prevalent precancer is overestimated.

Many studies suggest that even experienced colposcopists are challenged at identifying the worst lesion on the cervix, using visual criteria [22, 23]. We have observed that totally normal, non-acetowhite epithelium only rarely harbours precancer, limiting the value of random biopsies [23]. In support of this, a longitudinal study of women with minor cytological abnormalities and normal colposcopy impression showed a low risk of CIN3 over multiple years [24]. But even subtle acetowhitening can represent precancer (including histologic CIN3) and taking multiple biopsies of acetowhite lesions rather than one biopsy of the ‘worst’ lesion, substantially increases the sensitivity of the colposcopy [17, 23]. We recently demonstrated that the increased sensitivity achieved by using multiple targeted biopsies is consistent across important risk strata like referral cytology and HPV status, but we also showed that the absolute yield of disease strongly differed by prior risk [23]. Together, these data allow tailoring colposcopy–biopsy protocols to women based on their age, cytology result, and HPV status. While increasing evidence supports multiple biopsies of acetowhite lesions as an effective way to improve detection of cervical precancers, confirmation of the best colposcopic biopsy strategy and its promulgation as an international standard remains a major issue.

Another controversial issue in colposcopy is the value of endocervical sampling, specifically endocervical curettage (ECC). There are good data showing low marginal gain in sensitivity for detection of precancer in younger women once ectocervical biopsies are taken, but for older women (or in the case of inadequate visualisation of the transformation zone), the use of ECC is more supported [25]. Some practitioners routinely add ECC to ectocervical biopsies, perhaps because of particular concern over hard-to-detect glandular lesions, while others rarely do. A large, randomised trial would be required to answer this question, given the relative rarity of precancer restricted to the endocervical canal; we know, however, of no plans to launch such an investigation.

Regardless of which colposcopy-biopsy protocol is chosen, it remains an issue how to research, teach, and evaluate colposcopy. Pathologists are able to exchange microscope slides and, similarly, it is common to rely on colpophotographs, i.e. static images used for teaching and evaluation of colposcopists [26]. With modern equipment, static images can be extremely clear and realistic. However, static images cannot show the dynamic changes in response to uptake of acetic acid, in addition to the obvious issue of incomplete visualisation of epithelium within the endocervical canal especially as women age. Images are widely used for teaching colposcopy and certainly represent a wide range of cervical abnormalities. Colposcopy videos and direct ‘apprenticeship’ in the clinic can overcome the limitations of static images, but are much harder to standardise, disseminate, and teach to a larger population of colposcopists in training.

Aids for colposcopists have been extensively researched and even submitted formally for clinical approval and commercial use [27]. So far, none has proven particularly useful, despite sophisticated image analysis and addition of non-visible spectra or electrical current. The accurate distinction of precancer from atypical metaplasia has not yet been resolved. One recent effort relies on the sensitivity and dynamics of acetowhitening, but is still to be proven as a viable clinical tool [28]. Another interesting technology for use during colposcopy is high-resolution microendoscopy, a technique to evaluate lesion severity by real-time analysis of cellular and nuclear features using a probe. This technique is under evaluation for use in both low-resource and high-resource settings [29].

The net result of inaccuracy in the colposcopic examination is that it yields imperfect risk stratification; in many instances, neither a positive nor a negative examination can be completely trusted. As a corollary, a major contemporary problem is the large fraction of women undergoing colposcopy who are not identified to have precancer requiring treatment, but whose risk profile based on preceding screening results precludes return to routine screening. Women frequently return to colposcopy clinics, with strings of repeated examinations at substantial societal expense and negative psychologic effects. This problem might worsen when the fraction of referred women sent because of persistent HPV test positivity (rather than abnormal cytologic findings) increases. We need somehow to increase the sensitivity of the colposcopic procedure, with resultant increased reassurance of negative examination/biopsies, permitting release from colposcopic surveillance. Taking more biopsies of even subtle acetowhite lesions apparently can increase colposcopic sensitivity. Improving the targeting of biopsies of the worst lesion on the cervix could achieve the same safety with reduced number of biopsies, but currently, there is no proven strategy that can accomplish this. On the other side, because colposcopy is imperfectly sensitive, when the positive predictive value of certain combinations of screening results is very high, treatment might be indicated regardless of colposcopic findings or biopsy results. An example would be that of a persistently HPV16-positive woman with high-grade squamous intraepithelial lesion (HSIL) cytology; a normal colposcopic examination in this context is likely to represent a missed incipient precancer diagnosis.

The colposcopic procedure is imperfect, but by no means useless, as suggested by some; in fact, it rightfully remains the reference standard of diagnosis of cervical cancer/precancer. Because colposcopes are expensive and not universally available, there is a long history of attempts to replace this procedure with something inexpensive and easy to use. Cervicography (off-site evaluation of a static image) and speculoscopy (using a special blue-white light source) are now of historic interest. Unmagnified visual inspection with acetic acid (VIA), however, represents a very current issue [30]. VIA has been recommended by some prominent groups as a screening or triage option; screen-and-treat protocols without histologic confirmation are sometimes employed. However, VIA shares many problems with colposcopy, with additional disadvantages of less magnification and generally less training. Its role is beyond this discussion, but VIA produces a highly imperfect estimate of cervical cancer risk and one could hope for eventual replacement by a more accurate method.

## Conclusion

Colposcopy or a derivative procedure will remain an important intermediate step in most cervical screening programmes for the foreseeable future. Dr Mario Sideri was among a small group of physician-researchers most actively involved in evaluating and improving this important clinical procedure. He was instrumental in encouraging people with different, and often strong, opinions about colposcopy practice and performance to work together for the common cause of improving teaching and the practice of colposcopy. We need more attention to this area, and his loss is being felt acutely.
